# The Impact of Morbid Obesity on In-hospital Outcomes after Revision Total Hip Arthroplasty: An Analysis of the National Inpatient Sample

**DOI:** 10.5435/JAAOSGlobal-D-22-00023

**Published:** 2022-08-05

**Authors:** Inaya Hajj Hussein, Abdul Kareem Zalikha, Zachary Crespi, Andrei Tuluca, Avianna E. Arapovic, Mouhanad M. El-Othmani

**Affiliations:** From the Department of Foundational Medical Studies, Oakland University William Beaumont School of Medicine, Auburn Hills, MI (Dr. Hajj Hussein); the Department of Orthopaedic Surgery and Sports Medicine, Detroit Medical Center, Detroit, MI (Dr. El-Othmani and Dr. Zalikha); the Central Michigan University College of Medicine, Saginaw, MI (Crespi, and Tuluca); and the Oakland University William Beaumont School of Medicine, Auburn Hills, MI (Arapovic).

## Abstract

**Introduction:**

There remain limited data on the effect of obesity on in-hospital outcomes after revision total hip arthroplasty (rTHA).

**Methods:**

Discharge data from the National Inpatient Sample were used to identify patients undergoing rTHA from 2006 to 2015. Propensity score analysis was done to analyze the effects of obesity and morbid obesity on in-hospital economic and complication outcomes after rTHA.

**Results:**

The estimated 460,297 rTHAs were done during the study period. Obese patients were more likely to suffer from any complication than not obese patients (41.44% versus 39.41%, *P* = 0.0085), and morbidly obese patients were more likely to suffer from any complication than obese patients (47.22% versus 41.44%, *P* < 0.0001). Obesity was associated with increased risk of postoperative anemia compared with not obese patients, while morbid obesity was associated with increased risk of postoperative anemia, hematoma/seroma, wound dehiscence, and postoperative infection (*P* < 0.05). Morbidly obese patients also had a significantly greater average length of stay (6.40 days) than obese (5.23 days) and not obese (5.37 days) patients (*P* < 0.0001).

**Discussion:**

Although both obesity and morbid obesity are associated with higher risk of in-hospital postoperative complications after rTHA, morbid obesity is a larger risk factor and is associated with a longer length of stay.

Although primary total hip arthroplasty (THA) has a well-reported track record of excellent outcomes, the number of patients requiring revision THA (rTHA) for reasons such as implant failure, metallosis, infection, and instability has steadily increased in recent decades.^1^ In 2014, a total of 50,220 rTHAs were done, and the incidence of the procedure is projected to continue rising between 43% and 70% by 2030.^1^ As the increasing number of revisions annually constitutes a substantial economic burden to the healthcare system, identifying and optimizing risk factors will prove critical to improving outcomes after rTHA.^2,3^

Obesity is a public health epidemic that has been identified as a risk factor for requiring revision arthroplasty.^4^ Although obesity is a known risk factor for worse outcomes after primary THA, there have been fewer studies assessing its effect on outcomes after rTHA, with different studies demonstrating conflicting results. Perka et al^5^ found no association between obesity and perioperative morbidity and mortality after rTHA. Similarly, in a study of 246 patients, Watts et al^6^ reported similar outcomes between morbidly obese and nonobese patients. By contrast, other small prospective cohort studies found increased rates of adverse events and worsened functional and pain-related outcomes in obese patients after rTHA.^7-9^ Overall, the literature lacks consensus regarding the complication profile and economic outcomes among obese and morbidly obese patients during the immediate in-hospital period after rTHA, presenting a valuable addressable opportunity for perioperative risk stratification and optimization for this subset of patients.

In that context, the purpose of this study was to (1) assess the trend in type of and reason for rTHA among obese and morbidly obese patients and (2) compare in-hospital medical and economic outcomes between nonobese, obese, and morbidly obese patients using a large national database over an extended duration.

## Methods

Retrospective analysis was done using discharge data from 2006 to the third quarter of 2015 from the National Inpatient Sample, which is part of the Hospital Cost and Utilization Project. This database incorporates and accounts for approximately 20% of inpatient stays within the United States and includes information such as patient demographics, charges, comorbidities, and perioperative outcomes.^10^ The International Classification of Disease, Ninth Revision, Clinical Modification, was used for procedure/diagnosis codes within the National Inpatient Sample during our timeline of interest.

Our study includes patients at least 40 years old who underwent rTHA for any reason. Reason for revision was accounted for the following International Classification of Disease codes: dislocation/instability (996.42), mechanical loosening (996.41), infection (996.66), implant failure (996.43), other mechanical problems (996.47), periprosthetic osteolysis (996.45), periprosthetic fracture (996.44), bearing surface wear (996.46), and other mechanical complications (996.49). The type of revision was also collected using the following codes: all components (00.70), acetabular implant (00.71), femoral implant (00.72), acetabular liner and/or femoral head only (00.73), arthrotomy for removal of prosthesis (80.05), and other, not otherwise specified (81.53).

Discharge weights, clusters, and strata were all accounted for as recommended by the Agency for Healthcare Research and Quality. Once rTHA patients were identified, they were stratified into one of the three groups: obese body mass index (BMI) (278.00), morbidly obese (278.01), and none of these diagnoses (referred to as “not obese”).

We performed inverse probability of treatment weighting, a validated method of balancing covariates that helps minimize the effect of confounding bias and adjusts for the severity of comorbidities.^11^ Propensity score weighting was done using the DuGoff et al^12^ method by weighting patient demographics, hospital characteristics, and comorbidities using the Elixhauser comorbidity index. By incorporating multiple different comorbidities, the Elixhauser comorbidity index is commonly used in large database research to properly assess patient comorbidities and has been shown to be superior to other comorbidity indices in controlling for potential confounding effects of preexisting diseases.^13^ We thus elected to use the inverse probability of treatment weighting/propensity score weighting incorporating the Elixhauser comorbidities, in addition to patient demographics and hospital characteristics, as the statistical method to control for confounding effects. This technique is advantageous because this model is successful in controlling for comorbidities without losing a large number of patients, given our three cohorts.

The variable “any complication” was used as a composite measure referring to any cardiac, respiratory, peripheral vascular disease, hematoma/seroma, wound dehiscence, postoperative infection, gastrointestinal, genitourinary, deep vein thrombosis, pulmonary embolism, or postoperative anemia complication. Patient demographics, type and reason for revision, immediate postoperative complications, in-hospital length of stay (LOS), disposition, and economic outcomes were comparatively analyzed using these weighted cohorts. Continuous and categorical data were analyzed using Student *t*-tests and univariate logistic regression, respectively. Statistical significance of the data was defined at *P*-value < 0.05. All statistical analyses were done using SAS 9.4 (SAS Institute) for Windows.

## Results

### Demographics and Comorbidity Data

A total of 460,297 rTHAs were done during the study period. From 2006 to 2015, there was an increase in the relative proportion of patients with obesity (5.68% to 10.14%) or morbid obesity (2.22% to 7.57%). Morbidly obese patients were statistically significantly younger (62.35 versus 64.37 versus 68.54, *P* < 0.0001) than obese and not obese patients, respectively. Female (62.58% versus 53.36% versus 56.87%, *P* < 0.0001) and African American (10.26% versus 7.66% versus 5.66%, *P* < 0.0001) patients constituted a larger proportion among morbidly obese than in obese and not obese, respectively. The demographics of the study population are given in Table 1, and comorbidity data are given in Table 2.

**Table 1:** Demographic and Hospital Factors Among the Study Population, Stratified by Body Mass Index

Factor	Not Obese (n = 400,974)	Obese (n = 35,117)	Morbidly Obese (n = 24,206)	*P*
Age of patient in yr-mean (standard error)	68.54 (0.08)	64.37 (0.14)	62.35 (0.15)	<0.0001
Elective admission				
Nonelective	114,244 (28.49%)	8,588 (24.45%)	7,366 (30.43%)	<0.0001
Elective	285,841 (71.29%)	26,489 (75.43%)	16,782 (69.33%)	
Biological sex of patient				
Male	172,950 (43.13%)	15,676 (44.64%)	9,057 (37.42%)	<0.0001
Female	228,024 (56.87%)	19,440 (53.36%)	15,149 (62.58%)	
Primary payor				
Medicare	262,084 (65.36%)	19,601 (55.82%)	12,971 (53.59%)	<0.0001
Medicaid	15,860 (3.96%)	1,469 (4.18%)	1,341 (5.54%)	
Private payor	108,030 (26.94%)	12,403 (35.32%)	8,785 (36.29%)	
Self-pay	2824 (0.70%)	230 (0.65%)	183 (0.76%)	
No charge	480 (0.12%)	<10 cases	<10 cases	
Other	10,970 (2.74%)	1,269 (3.61%)	803 (3.32%)	
Unknown	727 (0.18%)	117 (0.33%)	80 (0.33%)	
Race of patient				
White	298,324 (74.40%)	25,370 (73.27%)	17,661 (72.96%)	<0.0001
African American	22,680 (5.66%)	2,688 (7.66%)	2,484 (10.26%)	
Hispanic	12,396 (3.09%)	1,296 (3.69%)	654 (2.70%)	
Asian or pacific islander	2,966 (0.74%)	212 (0.60%)	70 (0.29%)	
Native American	1,543 (0.38%)	102 (0.29%)	120 (0.50%)	
Other or unknown	63,065 (15.73%)	5,088 (14.49%)	3,217 (13.29%)	
Year of discharge				
2006	35,034 (8.74%)	2,160 (6.15%)	843 (3.48%)	<0.0001
2007	37,075 (2.95%)	2,191 (6.24%)	1,102 (4.55%)	
2008	39,243 (9.79%)	2,849 (8.11%)	1,596 (6.60%)	
2009	37,808 (9.43%)	2,766 (7.88%)	1,767 (7.30%)	
2010	41,194 (10.27%)	2,805 (7.99%)	2,494 (10.30%)	
2011	44,980 (11.22%)	3,876 (11.04%)	3,218 (13.30%)	
2012	43,230 (10.78%)	4,400 (12.53%)	2,900 (11.98%)	
2013	45,110 (11.25%)	4,955 (14.11%)	3,420 (14.13%)	
2014	45,030 (11.23%)	5,140 (14.64%)	3,895 (16.09%)	
2015	32,270 (8.05%)	3,975 (11.32%)	2,970 (12.27%)	
Bedsize of hospital				
Small	65,044 (16.22%)	5,817 (16.57%)	4,189 (17.30%)	0.0375
Medium	95,687 (23.86%)	9,097 (25.91%)	5,447 (22.50%)	
Large	238,109 (59.38%)	20,074 (57.16%)	14,494 (59.88%)	
Unknown	2,134 (0.53%)	128 (0.37%)	76 (0.31%)	
Location/teaching status				
Rural	28,180 (7.03%)	2,150 (6.12%)	1,509 (6.23%)	<0.0001
Urban nonteaching	143,218 (35.72%)	11,344 (32.30%)	8,007 (33.08%)	
Urban teaching	227,442 (56.72%)	21,495 (61.21%)	14,614 (60.37%)	
Unknown	2,134 (0.53%)	128 (0.37%)	76 (0.31%)	
Region of hospital				
Northeast	72,122 (17.99%)	5,872 (16.72%)	4,457 (18.41%)	<0.0001
Midwest	98,410 (24.54%)	9,692 (27.60%)	7,718 (31.89%)	
South	144,753 (36.10%)	12,036 (34.27%)	7,914 (32.69%)	
West	85,688 (21.37%)	7,516 (21.40%)	4,117 (17.01%)	

**Table 2:** Comparative Elixhauser Comorbidities Among Not Obese, Obese, and Morbidly Obese Patients

Factor	Not Obese (n = 400,974)	Obese (n = 35,117)	Morbidly Obese (n = 24,206)	*P*
Acquired immune deficiency syndrome (AIDS)	640 (0.16%)	<10 cases	<10 cases	0.1790
Alcohol abuse	9,816 (2.45%)	824 (2.35%)	335 (1.38%)	<0.0001
Deficiency anemias	70,229 (17.51%)	6,186 (17.61%)	4,550 (18.80%)	0.0963
Rheumatoid arthritis/collagen vascular disease	27,602 (6.88%)	2,289 (6.52%)	1,443 (5.96%)	0.0364
Chronic blood loss anemias	9,295 (2.32%)	710 (2.02%)	431 (2.19%)	0.2831
Congestive heart failure	24,152 (6.02%)	1,942 (5.53%)	1,898 (7.84%)	<0.0001
Chronic pulmonary disease	67,859 (16.92%)	7,093 (20.20%)	5,765 (23.82%)	<0.0001
Coagulopathy	16,292 (4.06%)	1,262 (3.59%)	901 (3.72%)	0.1021
Depression	57,911 (14.44%)	6,781 (19.31%)	5,205 (21.50%)	<0.0001
Uncomplicated diabetes	54,259 (13.53%)	8,041 (22.90%)	7,343 (30.34%)	<0.0001
Complicated diabetes	6,718 (1.68%)	1,307 (3.72%)	1,084 (4.48%)	<0.0001
Drug abuse	5,708 (1.42%)	594 (1.69%)	394 (1.63%)	0.1212
Hypertension	239,561 (59.74%)	25,570 (72.81%)	18,197 (75.17%)	<0.0001
Hypothyroidism	59,399 (14.81%)	5,781 (16.46%)	4,399 (18.18%)	<0.0001
Liver disease	8,051 (2.01%)	758 (2.16%)	555 (2.29%)	0.3022
Lymphoma	2,261 (0.56%)	83 (0.24%)	111 (0.46%)	0.0009
Fluid and electrolyte disorders	63,133 (15.74%)	5,303 (15.10%)	4,830 (19.95%)	<0.0001
Metastatic cancer	2,240 (0.56%)	133 (0.38%)	58 (0.24%)	0.0024
Other neurological disorders	28,123 (7.01%)	1,792 (5.10%)	1,273 (5.26%)	<0.0001
Paralysis	3,848 (0.96%)	276 (0.79%)	137 (0.56%)	0.0092
Peripheral vascular disorders	13,677 (3.41%)	1,023 (2.91%)	789 (3.26%)	0.0981
Psychoses	12,344 (3.08%)	1,471 (4.19%)	1,073 (4.43%)	<0.0001
Pulmonary circulation disorders	6,716 (1.68%)	551 (1.57%)	630 (2.60%)	<0.0001
Renal failure	27,955 (6.97%)	2,931 (8.35%)	2,407 (9.04%)	<0.0001
Solid tumor without metastasis	3,028 (0.76%)	175 (0.50%)	64 (0.27%)	<0.0001
Peptic ulcer disease excluding bleeding	96 (0.02%)	<10 cases	<10 cases	0.7010
Valvular disease	20,709 (5.17%)	1,557 (4.44%)	866 (3.58%)	<0.0001
Weight loss	12,123 (3.02%)	807 (2.30%)	703 (2.90%)	0.0034

### Type of and Reason for Revision

Across all three groups, the most common revision was revision of all components. A statistically significant difference was observed in the proportion of each revision between groups across all revisions. Information related to the type of rTHA is given in Table 3.

**Table 3:** Type of Revision Stratified by Not Obese, Obese, and Morbidly Obese

Factor	Not Obese (n = 400,974)	Obese (n = 35,117)	Morbidly Obese (n = 24,206)	*P*
All components	169,743 (42.33%)	15,046 (42.84%)	9,590 (39.62%)	0.0010
Acetabular implant	57,589 (14.36%)	4,886 (13.91%)	3,021 (12.48%)	0.0011
Femoral implant	62,808 (15.66%)	5,047 (14.37%)	3,711 (15.33%)	0.0195
Acetabular liner and/or femoral head only	57,875 (14.43%)	5,608 (15.97%)	3,620 (14.58%)	0.0025
Arthrotomy for removal of prosthesis	36,422 (9.08%)	3,598 (10.25%)	3,688 (15.23%)	<0.0001
Other, not otherwise specified	20,592 (5.14%)	1,316 (3.75%)	900 (3.72%)	<0.0001

There was a notable difference in the rates of reason for revision between all groups, and the top three reasons for revision among all groups were dislocation/instability, mechanical loosening, and infection. Interestingly, infection was the most common reason for obese and morbidly obese, while dislocation/instability was the most common for the not obese cohort. The reason for revision for all groups is given in Table 4.

**Table 4:** Reason for Revision Stratified by Not Obese, Obese, and Morbidly Obese

Factor	Not Obese (n = 400,974)	Obese (n = 35,117)	Morbidly Obese (n = 24,206)	*P*
Dislocation/instability	87,605 (21.85%)	6,538 (18.62%)	3,957 (16.35%)	<0.0001
Mechanical loosening	79,156 (19.74%)	6,603 (18.80%)	4,200 (17.35%)	0.0002
Infection	60,562 (15.10%)	6,806 (19.38%)	6,777 (28.00%)	<0.0001
Implant failure	23,103 (5.76%)	1,681 (4.79%)	1,107 (4.57%)	<0.0001
Other mechanical problems	53,802 (13.42%)	5,353 (15.24%)	3,090 (12.77%)	0.0001
Periprosthetic osteolysis	27,668 (6.90%)	2,350 (6.69%)	1,051 (3.43%)	<0.0001
Periprosthetic fracture	26,332 (6.57%)	1,997 (5.69%)	1,571 (6.49%)	0.0163
Bearing surface wear	22,306 (5.56%)	2,038 (5.80%)	961 (3.97%)	<0.0001
Other mechanical complications	15,882 (3.96%)	1,263 (3.60%)	752 (3.11%)	0.0051

### In-hospital Complications

Obese patients were more likely to suffer from any complication than not obese patients (41.44% versus 39.41%, *P* = 0.0085). When analyzing individual complications, obese patients were more likely than not obese patients to suffer from postoperative anemia (37.67% versus 35.67%, *P* = 0.0081). Not obese patients were more likely than obese patients to endure cardiac complications (0.93% versus 0.59%, *P* = 0.0054) and to die during hospitalization (0.75% versus 0.34%, *P* = 0.0001). These differences and the overall complications assessed are reflected in Table 5.

**Table 5:** Inverse Probability of Treatment Weighting Outcomes Analysis Comparing Not Obese and Obese Patients

Factor	Not Obese	Obese	OR (95% CI)	*P*
Any complications	39.41%	41.44%	1.08 (1.02-1.14)	0.0085
Central nervous system (CNS) complication	0.20%	0.16%	0.82 (0.46-1.48)	0.5211
Cardiac complication	0.93%	0.59%	0.65 (0.48-0.88)	0.0054
Peripheral vascular disease (PVD) complication	0.09%	0.09%	1.04 (0.48-2.28)	0.9163
Respiratory complication	0.46%	0.38%	0.83 (0.57-1.20)	0.3146
Gastrointestinal (GI) complication	0.37%	0.34%	0.92 (0.62-1.38)	0.6969
Genitourinary (GU) complication	0.58%	0.46%	0.81 (0.56-1.17)	0.2565
Hematoma/seroma	2.87%	3.25%	1.14 (0.99-1.31)	0.0658
Wound dehiscence	1.04%	1.30%	1.19 (0.96-1.49)	0.1190
Postoperative infection	1.11%	1.31%	1.19 (0.95-1.49)	0.1312
Deep vein thrombosis (DVT)	0.74%	0.61%	0.83 (0.62-1.12)	0.2260
Pulmonary embolism (PE)	0.40%	0.47%	1.03 (0.71-1.50)	0.8667
Postoperative anemia	35.67%	37.67%	1.08 (1.02-1.15)	0.0081
Died during hospitalization	0.75%	0.34%	0.44 (0.30-0.66)	0.0001
Length of stay (days)	5.37	5.27	—	0.0840
Total charges ($)	$75,158	$78,891	—	0.0008

Morbidly obese patients were more likely to suffer from any complications than not obese patients (47.22% versus 39.41%; *P* < 0.0001). For individual complications, morbidly obese patients were more likely than not obese patients to endure hematoma/seroma (4.34% versus 2.87%; *P* < 0.0001), wound dehiscence (2.21% versus 1.11%; *P* < 0.0001), postoperative infection (2.28% versus 1.11%; *P* < 0.0001), and postoperative anemia (42.29% versus 35.67%; *P* < 0.0001). These differences and the overall data set are given in Table 6.

**Table 6:** Inverse Probability of Treatment Weighting Outcomes Analysis Comparing Not Obese and Morbidly Obese Patients

Factor	Not Obese	Morbidly Obese	OR (95% CI)	*P*
Any complications	39.41%	47.22%	1.37 (1.29-1.46)	<0.0001
Central nervous system (CNS) complication	0.20%	0.25%	1.18 (0.66-2.13)	0.5720
Cardiac complication	0.93%	0.76%	0.80 (0.57-1.11)	0.1755
Peripheral vascular disease (PVD) complication	0.09%	0.10%	1.14 (0.46-2.83)	0.7724
Respiratory complication	0.46%	0.32%	0.70 (0.42-1.15)	0.1576
Gastrointestinal (GI) complication	0.37%	0.22%	0.64 (0.36-1.14)	0.1265
Genitourinary (GU) complication	0.58%	0.46%	0.75 (0.49-1.17)	0.2072
Hematoma/seroma	2.87%	4.34%	1.55 (1.34-1.79)	<0.0001
Wound dehiscence	1.04%	2.21%	2.11 (1.73-2.58)	<0.0001
Postoperative infection	1.11%	2.28%	2.08 (1.69-2.56)	<0.0001
Deep vein thrombosis (DVT)	0.74%	0.96%	1.29 (0.96-1.73)	0.0957
Pulmonary embolism (PE)	0.40%	0.40%	0.98 (0.63-1.54)	0.9414
Postoperative anemia	35.67%	42.29%	1.32 (1.24-1.41)	<0.0001
Died during hospitalization	0.75%	0.57%	0.75 (0.52-1.09)	0.1314
Length of stay (d)	5.37	6.43	—	<0.0001
Total charges ($)	$75,158	$88,426	—	<0.0001

Morbidly obese patients were more likely to suffer from any complication than obese patients (47.22% versus 41.44%, *P* < 0.0001). Morbidly obese patients were more likely than obese patients to endure hematoma/seroma (4.34% versus 3.25%, *P* = 0.0014), wound dehiscence (2.21% versus 1.30%, *P* = 0.0001), postoperative infection (2.28% versus 1.31%, *P* = 0.0001), deep vein thrombosis (0.96% versus 0.61%; *P* = 0.0374), and postoperative anemia (42.29% versus 37.67%; *P* < 0.0001). These differences and the overall data set are presented in Table 7.

**Table 7:** Inverse Probability of Treatment Weighting Outcomes Analysis Comparing Obese and Morbidly Obese Patients

Factor	Obese	Morbidly Obese	OR (95% CI)	*P*
Any complications	41.44%	47.22%	1.27 (1.17-1.38)	<0.0001
Central nervous system (CNS) complication	0.16%	0.25%	1.44 (0.66-3.12)	0.3610
Cardiac complication	0.59%	0.76%	1.23 (0.79-1.91)	0.3517
Peripheral vascular disease (PVD) complication	0.09%	0.10%	1.10 (0.35-3.46)	0.8751
Respiratory complication	0.38%	0.32%	0.85 (0.46-1.56)	0.5916
Gastrointestinal (GI) complication	0.34%	0.22%	0.69 (0.35-1.36)	0.2861
Genitourinary (GU) complication	0.46%	0.46%	0.93 (0.54-1.61)	0.8075
Hematoma/seroma	3.25%	4.34%	1.36 (1.13-1.64)	0.0014
Wound dehiscence	1.30%	2.21%	1.77 (1.34-2.35)	0.0001
Postoperative infection	1.31%	2.28%	1.75 (1.31-2.33)	0.0001
Deep vein thrombosis (DVT)	0.61%	0.96%	1.54 (1.03-2.32)	0.0374
Pulmonary embolism (PE)	0.47%	0.40%	0.95 (0.55-1.65)	0.8625
Postoperative anemia	37.67%	42.29%	1.22 (1.12-1.32)	<0.0001
Died during hospitalization	0.34%	0.57%	1.70 (0.99-2.90)	0.0533
Length of stay (d)	5.27	6.43	—	<0.0001
Total charges ($)	$78,891	$88,426	—	<0.0001

### Resources Utilization

Morbidly obese patients had a significantly greater average LOS (6.43 days) than obese (5.27 days) and not obese (5.37 days) patients (*P* < 0.0001). Morbidly obese patients also had significantly higher total charges ($88,426) than obese ($78,891) and not obese ($75,158) patients. A notable difference was observed in total charges, but not LOS, between the obese and not obese groups.

## Discussion

Obesity puts patients at increased risk of perioperative complications, revision, and overall worse outcomes after primary THA.^14-16^ As such, we sought to comparatively examine in-hospital outcomes and the etiology and type of revision between not obese, obese, and morbidly obese patients undergoing rTHA. This study noted markedly worse outcomes for morbidly obese patients and notable differences in the type of and reason for revision between the three groups.

This study found that in the in-hospital, postoperative period, both obese and morbidly obese patients were markedly more likely to experience any complication when compared with the not obese group, with markedly worse odds for the morbidly obese than the obese group. Comparatively, previous studies reported conflicting effects of BMI on outcomes after rTHA, with some studies noting no association between obesity and postoperative morbidity and mortality, while others reporting worse outcomes and increased complications among obese patients.^7-9,17^ Perhaps most notably, a larger database study of 18,866 patients by Roth et al^18^ demonstrated a J-shaped curve between the relationship of increasing BMI and 30-day complications, with the lowest rate of complications occurring around a BMI of 30, with increasing complications as BMI increased. Overall, this study reinforces the mounting evidence suggesting that morbid obesity is a greater risk factor than obesity for adverse outcomes after rTHA.

The increased complications of postoperative hematoma and seroma formation, wound dehiscence, early infection, and postoperative anemia noted in the morbidly obese cohort in this study are intuitive with the understanding that increasing BMI increases the complexity of rTHA. The larger amount of adipose tissue may predispose to increased dead space and consequent hematoma and fluid collection, which in itself may predispose to wound complications, infection, and anemia. In addition, obese patients are at risk for paradoxical micronutrient and macronutrient deficiencies, further increasing their theoretical potential for wound complications.^19^ It is noteworthy that our analysis revealed a markedly higher risk for these complications among morbidly obese, but not obese patients. It is likely that there is a threshold of increasing BMI where these physiologic explanations become clinically evident in observed wound complications in morbidly obese patients.^20^ In addition, as the procedure becomes more demanding, surgical time is often increased, and increased blood loss associated with technical challenges and increased dissection through hypertrophic subcutaneous tissue can further contribute to postoperative anemia and wound complications.

Regarding economic outcomes, this study reported markedly increased LOS and total charges among the morbidly obese when compared with nonobese patients, while there was a notable difference only in total charges among obese patients. The increased LOS found in morbidly obese patients mimics several complication findings, which is understandable given that increased in-hospital medical complications have been shown to be associated with longer LOS.^21^ As rTHA places increasing strain on the healthcare system, safely decreasing LOS is a critical target for procedure-level cost-containment.^22^ The increased LOS that morbidly obese patients face should serve as a relevant quality control target because improved perioperative management protocols are developed for these patients.

Among all three groups, revision of all components was the most common type of procedure performed. Regarding reason for revision, morbidly obese patients were markedly more likely to require revision due to infection than obese patients, who in turn had a similar higher likelihood than the not obese group. These findings are concordant with previous studies, which noted obesity as a risk factor for periprosthetic infection, and morbid obesity even further increases this risk.^23,24^ Several potential explanations for this increased risk have been provided, including greater technical difficulty and longer duration of surgery, poor vascularity of adipose tissue, and other underlying comorbidities.^24^ This knowledge should help inform preoperative preventive processes because it is imperative that obese and morbidly obese patients be counseled and optimized for their uniquely increased risk of infection and revision after primary arthroplasty.

Several of the limitations of this study are inherent to large database studies. Although large national databases provide high volumes of data, disadvantages such as missing data and erroneous data exist.^25^ Despite this risk for errors, comorbidity and complication data in administrative databases have been validated as accurate.^26^ Another limitation of this study was that complications and outcomes were limited to the immediate in-hospital setting, which precluded the analysis of outcomes or complications that may occur after discharge. Although it is imperative to evaluate the long-term complications in the patient population we analyzed, to provide sound conclusions that respect the confinements of the database, this study focused primarily on immediate in-patient outcomes, resource utilizations, and complications, which themselves provide valuable information and quality control targets.

Despite these limitations, this study had several important strengths in design and scientific contribution. To the best of the authors' knowledge, this is the largest study of its kind to demonstrate increased rates of adverse postoperative complications and worse economic outcomes in morbidly obese patients in the critical in-hospital postoperative period after rTHA. The uniquely increased risk for adverse outcomes faced by morbidly obese patients is important to frame patient discussions and for consideration when designing perioperative management protocols for these patients. In addition, the findings of this study were strengthened by its propensity weighting statistical methodology, which allowed for controlling for a large number of potentially confounding demographic and medical comorbidities.

In conclusion, this study demonstrated that although both obesity and morbid obesity are risk factors for adverse in-hospital complication outcomes after rTHA, morbid obesity is a markedly greater risk factor for many adverse complications and increased LOS and total charges. As the obesity epidemic burgeons globally, and as the rates of rTHA continue to increase, understanding these different risks is critical to frame preoperative patient discussions and for perioperative management and optimization planning. Future attention should be directed to establishing preoperative targets and improving perioperative management protocols to improve outcomes in this increasing subset of patients.
